# Tart Cherry Concentrate Does Not Alter the Gut Microbiome, Glycaemic Control or Systemic Inflammation in a Middle-Aged Population

**DOI:** 10.3390/nu11051063

**Published:** 2019-05-13

**Authors:** Rebecca Lear, Mary O’Leary, Lee O’Brien Andersen, Corey Carrington Holt, Christen Rune Stensvold, Mark van der Giezen, Joanna L. Bowtell

**Affiliations:** 1 Department of Sport and Health Sciences, College of Life and Environmental Sciences, University of Exeter, St. Luke’s Campus, EX1 2LU Exeter, UK; rl442@exeter.ac.uk (R.L.); m.oleary@exeter.ac.uk (M.O.); 2 Laboratory of Parasitology, Department of Bacteria, Parasites & Fungi, Infectious Disease Preparedness, Statens Serum Institut, Artillerivej 5, 2300 Copenhagen S, Denmark; OBI@ssi.dk (L.O.A.); RUN@ssi.dk (C.R.S.); 3 Biosciences, University of Exeter, EX1 2LU Exeter, UK; corey.holt@cefas.co.uk (C.C.H.); m.vandergiezen@exeter.ac.uk (M.v.d.G.)

**Keywords:** polyphenol, Montmorency, cherry, microbiome

## Abstract

Limited evidence suggests that the consumption of polyphenols may improve glycaemic control and insulin sensitivity. The gut microbiome produces phenolic metabolites and increases their bioavailability. A handful of studies have suggested that polyphenol consumption alters gut microbiome composition. There are no data available investigating such effects in polyphenol-rich Montmorency cherry (MC) supplementation. A total of 28 participants (aged 40–60 years) were randomized to receive daily MC or glucose and energy-matched placebo supplementation for 4 wk. Faecal and blood samples were obtained at baseline and at 4 wk. There was no clear effect of supplementation on glucose handling (Homeostatic Model Assessment of Insulin Resistance (HOMA-IR) and Gutt indices), although the Matsuda index decreased significantly in the MC group post-supplementation, reflecting an increase in serum insulin concentration. Contrastingly, placebo, but not MC supplementation induced a 6% increase in the Oral Glucose Insulin Sensitivity (OGIS) estimate of glucose clearance. Serum IL-6 and C reactive protein were unaltered by either supplement. The faecal bacterial microbiome was sequenced; species richness and diversity were unchanged by MC or placebo and no significant correlation existed between changes in *Bacteroides* and *Faecalibacterium* abundance and any index of insulin sensitivity. Therefore, 4 weeks of MC supplementation did not alter the gut microbiome, glycaemic control or systemic concentrations of IL-6 and CRP in a middle-aged population.

## 1. Introduction

The World Health Organisation (WHO) estimates that in 2016, 39% of the global adult population were overweight (BMI >25 kg.m^−2^) and 13% were obese (BMI >30 kg.m^−2^). Globally, Type 2 diabetes (T2D) prevalence was estimated at 382 million in 2013 and is projected to rise to 592 million by 2035 [1]. Therefore, strategies that enhance insulin sensitivity and metabolic function must be developed in order to reduce the population incidence of the metabolic syndrome and T2D. Epidemiological evidence suggests that the consumption of polyphenols and anthocyanins in particular may reduce the risk of developing T2D, independent of body weight and body mass index (BMI) [2]. There is some direct evidence that chronic supplementation with fruit polyphenols improves insulin sensitivity. Stull et al. demonstrated, via hyperinsulinaemic euglycaemic clamps, increased insulin sensitivity following 6 weeks of 45 g.d^−1^ (1462 mg of total phenolics, 668 mg of anthocyanins per day) blueberry powder supplementation [3]. To date, only a handful of randomised controlled human studies of glucose metabolism and chronic polyphenol supplementation have been performed and the results are conflicting, with phenolic and anthocyanin doses being rarely reported [4]. There is therefore a need to perform well designed, suitably powered human randomised controlled trials to address these questions using dynamic measures of insulin sensitivity such as the oral glucose tolerance test (OGTT). MC contains a high concentration of parent flavonoids and phenolic acids (Phenol-Explorer Database [5]), but there are no such published studies on Montmorency cherry (MC) supplementation.

A number of cellular and molecular mechanisms are postulated to mediate the promotion of glucose tolerance by polyphenol supplementation, with such processes occurring in a variety of tissues, including skeletal muscle, adipose tissue, pancreas and liver. Postulated mechanisms include inhibition of α-amylase and α-glucosidase activities, enhanced glucose transporter 4 expression and translocation to the cell membrane; for a review, see [4]. However, >90% of polyphenols pass through the small intestine, reaching the large intestine where microbiota produce phenolic metabolites that have been elegantly shown to peak systemically 24 h after ingestion [6]. Therefore, the modulation of parent polyphenols by the gut microbiome to produce phenolic metabolites and increase their bioavailability is a mechanistic consideration that exists upstream of molecular changes in tissues that are traditionally thought to modulate glucose disposal. Such phenolic metabolites may act directly on tissues to enhance glucose disposal. Epigallocatechin-3-O-gallate (EGCG) is protective of insulin sensitivity in rat L6 muscle cells treated with the insulin resistance-inducing drug dexamethasone [7] and cinnamon-derived polyphenols promote the mRNA and protein expression of insulin signalling and glucose transport pathway components in 3T3-L1 adipocytes [8].

In addition to the gut microbiome altering polyphenolic metabolite availability for their direct systemic action on insulin sensitive tissues, polyphenol supplementation has also been associated with reductions in systemic inflammation. Chronic inflammation is an important aetiological factor in insulin resistance [9,10,11]. MC consumption has been shown to induce anti-oxidant and anti-inflammatory effects in healthy people [12] and to reduce oxidative damage and inflammatory responses to the oxidative stress of intensive exercise [13,14,15] and ischaemia reperfusion injury [16]. Such decreases in inflammation may be gut-derived; prebiotic treatment in obese mice increased *Bifidobacterium* spp and *Lactobacillus* spp, decreased systemic inflammation and this was associated with increased endogenous production of GLP-2 and improved gut barrier integrity [17]. Systemic inflammation may also be reduced by phenolic metabolites acting directly on peripheral tissues. Phenolic supplementation can reduce adipocyte [18,19], muscle [19] and hepatic [20,21] expression of pro-inflammatory cytokines. However, the phenolic sources and the doses used have varied across studies. For example, black soybean seed coat, providing circa 20 mg total procyanidin/d and reduced mesenteric adipose tissue TNFα gene expression approximately 10-fold in male C57BL/6 mice fed a 30% fat diet for 14 weeks [18]. A grape seed extract containing circa 9.6 mg total procyanidins reduced mesenteric adipose tissue TNFα gene expression approximately 5-fold in female Wistar rats fed a 60% fat diet for 19 weeks [19]. Rodent studies have suggested that increased *Bifidobacterium* spp is associated with reductions in inflammatory markers such as IL6, IL-1α, IL-1β, TNFα, and MCP1 [17,22,23] and improved glucose tolerance [22]. However, there are only a handful of published human intervention studies that have examined the effect of polyphenol consumption on the gut microbiome. Such studies suggest that *Lactobacillus* spp [24], *Bacteroides* spp [25] and *Bifidobacterium* spp [24,25,26,27] may increase following polyphenol consumption, with some studies describing parallel anti-inflammatory effects [24,25]. Such studies have largely used polyphenolic extracts (e.g., grape [26]) and powders (e.g., blueberry, cocoa [24,27]) that provided <15 g of sugars per day. There are currently no data available on the effects of Montmorency cherry supplementation on the gut microbiome or glucose tolerance. We hypothesised that 4 weeks of MC supplementation would improve blood glucose control and reduce biomarkers of systemic inflammation in middle-aged men and women, and that these adaptations may be accompanied by changes in the gut microbiome.

## 2. Materials and Methods

### 2.1. Participant Recruitment

This study was approved by the University of Exeter’s Sport and Health Sciences Research Ethics Committee and conformed with the principles detailed in the Declaration of Helsinki. All participants gave written informed consent to participate. A total of 30 physically untrained and non-obese middle-aged men and women (40–60 years) were screened and recruited; 28 participants completed the study. One participant completed the screening visit but was unable to attend for the test visits due to personal circumstances; the second completed the first baseline assessment but withdrew shortly thereafter. Both had been randomised to the MC group. Participants were pre-screened by telephone or email for self-reported inclusion/exclusion criteria. Potential participants who had, in the last 6 months, taken antibiotics, medications that alter metabolic or gastrointestinal function, prebiotics or probiotics, or who had a history of chronic constipation, diarrhoea or any other gastrointestinal problems were excluded. Participants were asked to refrain from taking any nutritional supplements for the duration of the study. Participants were pair-matched on the basis of sex, physical activity and BMI and then randomized to condition. Sample size calculations were based on a hypothesised increase in *Bifidobacterium* with MC supplementation. The estimated magnitude of change was determined from published research, in which the effects of 6 weeks of blueberry supplementation on the gut microbiome was assessed [27]. With greater than 90% power, 5% significance, within participant SD 0.8, and a sample size of 15 participants per group, we estimated that we would be able to detect an effect size difference in *Bifidobacterium* content of 1.2.

### 2.2. Experimental Design

A double-blind, randomised controlled study was conducted. Participants attended for a visit during which informed consent was obtained and the EPIC-Norfolk food frequency questionnaire [28] and the single-item physical activity questionnaire [29] were completed. Body weight and height and waist circumference were measured. Waist circumference was measured in the horizontal plane midway between lowest rib and the iliac crest [30]. Participants then completed a 3-day weighed food diary and wore a triaxial accelerometer for 3 days prior to the first test visit in order to obtain a baseline value for replication. Participants were invited back to the laboratory for the first test visit after an overnight fast and provided a faecal sample. Participants rested in a supine position in a dimly lit, temperature-controlled room for 10 min, after which indirect calorimetry measures were taken and a cannula was inserted into the antecubital vein. A basal blood sample was collected, and a standard oral glucose tolerance test was completed. An oral glucose tolerance test (OGTT) was administered [31]; participants were given a drink containing 75 g glucose in a 25% solution, and asked to rest quietly for the following 2 h, with indirect calorimetry measures and blood sample collection taking place every 15 min. Participants were randomised to receive MC or a visually similar cherry flavoured placebo and were given 4 bottles of supplement (420 mL), each containing a sufficient supply for one week, and labelled week 1 to 4. Participants were asked to consume 30 mL every morning and evening for the 4-week period totalling 60 mL per day. This is an identical dosing strategy to that used in studies that have shown MC enhances recovery from exercise [15,32,33]. Compliance was self-reported. Participants consumed a daily dose of MC that provided 296 mg total anthocyanins and 1040 mg total polyphenols (determined via batch analysis, Atlas Bioscience Labs) or an equivalent volume of glucose and energy-matched placebo each providing 49 g carbohydrate. On 3 days of each week of the 4-week supplementation period, participants completed a weighed food diary and wore a triaxial accelerometer; 2 days of data collection is considered sufficient for moderate to vigorous physical activity (MVPA) quantification [34]. Following 4 weeks of supplementation, participants were asked to replicate their baseline food intake and physical activity and then return to the lab for the post-supplementation visit where the same procedures were followed.

### 2.3. Dietary and Physical Activity Analysis

Participants completed weighed food diaries during the study protocol, and an EPIC-Norfolk food frequency questionnaire was completed at their first visit. The diaries were analysed using Nutritics nutritional analysis software. The EPIC-Norfolk was used to calculate habitual flavonoid, anthocyanin and proanthocyanin intake; this is a well validated approach [35,36,37]. Physical activity was assessed using triaxial accelerometers (ActivInsights Ltd., Kimbolton, Cambridgeshire, United Kingdom) placed on the left wrist of participants and worn continuously for the same periods as the food diaries. Triaxial acceleration was measured between ±8 g, at 100 Hz, and data were downloaded after each collection period to a personal computer according to manufacturer’s instructions and raw triaxial acceleration values were converted to resultant acceleration. Data were averaged over 5 s-epochs and previously validated acceleration cutpoints were applied to identify sedentary time and time spent performing light, moderate and vigorous physical activity [38]. Metabolic inflexibility—the impaired capacity of the body to match fuel oxidation to fuel availability [39]—is often a feature of impaired glucose tolerance [40]. Therefore, given our interest in the effect of MC on glucose handling, we measured oxygen consumption and carbon dioxide production by pulmonary gas analysis (Cortex MetaLyzer 3B, Cortex Biophysik GmbH, Leipzig, Germany), and standard indirect calorimetry equations were applied to calculate fat and carbohydrate oxidation rate and respiratory exchange ratio (RER) [41].

### 2.4. Blood Sampling and Analysis

Blood samples were obtained via cannulation of the antecubital vein. Whole blood glucose concentration was measured immediately in sodium fluoride vacutainers using a glucose and lactate analyser (2900 Series Analyser, YSI, Yellow Springs, OH, USA). Serum separator tubes were maintained at room temperature for 30 min and centrifuged at 4000 rpm for 15 min at 4 °C. The resulting serum samples for further analysis were stored at −80 °C. Plasma hsCRP concentration was measured via immunoturbidimetric method using a Roche P800 analyser (Roche 800, Mannheim, Germany). Serum insulin concentration was measured using a commercially available immunoassay (ProQuantum, Thermo Fisher, Waltham, MA, USA). Plasma IL-6 concentration was measured using a commercially available ELISA kit (Quantikine, R&D Systems, Minneapolis, MN, USA). The Homeostatic Model Assessment of Insulin Resistance (HOMA-IR), The Matsuda, The Gutt and the Oral Glucose Insulin Sensitivity (OGIS) indices were calculated according to established formulae [42].

### 2.5. Faecal Microbiome Analysis

Faecal samples were collected at baseline and post-4-week MC supplementation using a stool nucleic acid collection tube (Norgen, Biotek Corporation, Ontario, Canada). Participants were asked to collect a sample in the 48 h preceding their visit to the lab. Collection tubes contained Norgen’s Stool Preservative in a liquid format, allowing 2 g stool samples to be stored at room temperature for up to 2 years until Genomic analysis. Genomic DNA was isolated from 200 mg stool using a commercially available kit (QIAamp DNA Stool Mini Kit, Qiagen, Hilden, Germany) according to the manufacturer’s instructions. DNA was quantified using a UV-Vis spectrophotometer (NanoDrop 2000 spectrophotometer (Thermo Scientific, Waltham, MA, USA).

The 16S rDNA gene was targeted for amplification, using a modified version of the published universal prokaryotic primers 341F/806R, targeting the V3-V4 hyper-variable regions (pmid 15696537) [43,44]. The forward primer had three additional nucleotides attached in the 5′ end (ACTCCTAYGGGRBGCASCAG, 341F3) and the reverse primer had five additional nucleotides attached in the 5′ end (AGCGTGGACTACNNGGGTATCTAAT, 806R5).

Purified genomic DNA from each sample was initially amplified using the 16S primers. For each primer, the rDNA was amplified using a short PCR setup: (1) Initial denaturation −95 °C for 3 min; (2) elongation—20 cycles of 95 °C for 30 sec, 60 °C for 1 min, and 72 °C for 30 sec; and (3) a final elongation at 72 °C for 7 min. PCR was performed in a 25 µl volume, using the Extract-N-Amp PCR ReadyMix (Sigma-Aldrich, St Louis, MO, USA) with 0.4 µM of each primer and 2 µl template. This amplification PCR (PCR1) was prepared for sequencing by a second PCR (PCR2 or adaptor PCR) using the same PCR program. PCR2 attaches an adaptor A, an index i5, and a forward sequencing primer site (FSP) in the 5′ end of the amplicons and an adaptor B, an index i7, and a reverse sequencing primer site (RSP) to the 3′ end of the amplicons. Hence, four different PCR amplicon products were generated for each sample. DNA was quantified using the Quant-ITTM dsDNA High Sensitivity Assay Kit (Thermo Fisher Scientific, Waltham, MA, USA), and PCR2 products were pooled in equimolar amounts between samples into the pooled amplicon library (PAL). Undesirable DNA amplicons were removed from the PAL by Agencourt AMPure XP bead (Beckman Coulter) purification in a two-step process. Firstly, DNA fragments below 300 nt were removed using a 10 µL PAL to 24 µL AMPure beads ratio, following the manufactures protocol and eluted in 40 µL TE buffer. Secondly, large DNA fragments above 1 kbp were removed using a 10 µL AM1 to 16 µL AMPure beads ratio. The resulting purified PAL was diluted to its final concentration of 11.5 pM DNA with a 0.001 N NaOH concentration, used for sequencing on the Illumina MiSeq desktop sequencer (Illumina Inc., San Diego, CA, USA). The library was sequenced with the 500-cycle MiSeq Reagent Kit V2 in a 2x250 nt setup (Illumina Inc., San Diego, CA, USA).

Data were analysed with BION (http://box.com/bion), a newly developed pipeline. The pipeline accepts raw sequence data and includes steps for de-multiplexing, primer-extraction, sampling, sequence- and quality-based trimming and filtering, de-replication, clustering, chimera-checking, reference data similarities and taxonomic mapping and formatting. Non-overlapping paired reads are allowed for analysis.

The filtered abundance matrix was analysed in R using the phyloseq (v.1.26.0) and ggplot (v.3.1.0) packages. Species richness and diversity were estimated with the plot_richness function using observed counts and the inverse of the Simpson’s diversity index. For plotting, abundance counts were agglomerated to a genus level, transformed to relative abundance and filtered to remove those representing <2% of the entire community. The dutchmasters package was used to create a colour palette. Principle Component Analysis using the Bray–Curtis measure of dissimilarity was computed with the plot_ordination function (seed set at 1). Significance of clustering was measured using the adonis function of the vegan package (v.2.5.3).

### 2.6. Statistical Analysis

Data were analysed in SPSS v 24 (IBM, Armonk, NY, USA). Two-way mixed model ANOVAs were used to identify the effects of cherry supplementation on the main outcome measures. Where possible, data were transformed to achieve normality. Outliers >3 standard deviations from the sample mean were eliminated and the Greenhouse–Geisser correction for violation of sphericity was used. Where a statistically significant interaction existed, simple main effects are reported. For changes in microbial profiles and metabolic function markers, data could not be transformed to achieve normality and Spearman’s rank correlation coefficients were calculated. *p* < 0.05 was accepted as the threshold for statistical significance. Data are expressed as mean ± SD.

## 3. Results

### 3.1. Indices of Glucose Tolerance and Insulin Sensitivity

**Figure 1:**
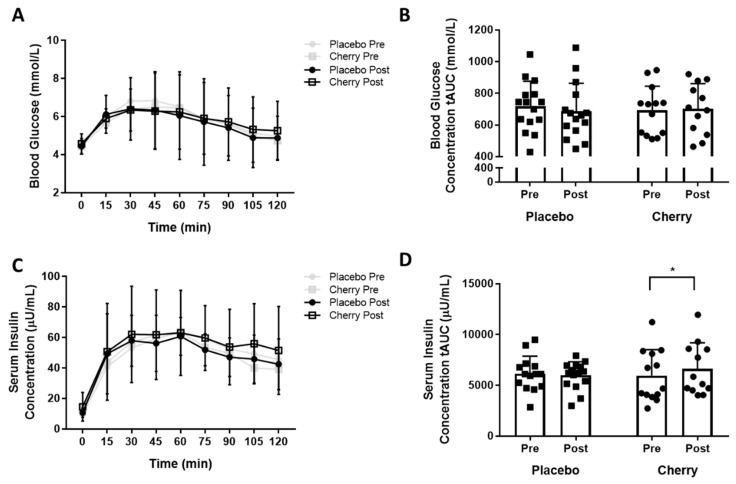
Systemic Glucose and Insulin Responses to an Oral Glucose Tolerance Test (OGTT) in Placebo and Montmorency Cherry Supplemented Middle-Aged Participants. A total of 28 non-obese middle-aged men and women (40–60 years) consumed a daily dose of Montmorency cherry concentrate (*n* = 13) or an equivalent volume of glucose and energy-matched placebo (*n* = 15) for 4 weeks. An oral glucose tolerance test was conducted pre- and post-supplementation. (**A**) Blood glucose concentrations during OGTT; (**B**) Blood glucose total area under the curve (tAUC) during OGTT; (**C**) Serum insulin concentrations during OGTT; (**D**) Serum insulin tAUC during OGTT. Missing data points are due to failure of venous access at a necessary timepoint. * *p* < 0.05. In Figure 1B,D, black squares = placebo, black circles = cherry.

Participant baseline characteristics are presented in Table 1. Participant groups were well matched, although the differences in age (placebo = 53.2 ± 5.6, MC = 48.9 ± 5.4, *p* = 0.05) and BMI (placebo 24.1 ± 2.1, MC = 25.8 ± 2.3, *p* = 0.05) did approach statistical significance. There was no statistically significant difference in mean blood glucose concentration between supplementation groups (Figure 1A) either pre- (F(1,25) = 0.166, *p* = 0.687, partial η^2^ = 0.007) or post- (F(1,25) = 0.081, *p* = 0.778, partial η^2^ = 0.003) supplementation. A main effect of time was noted both pre- (F(2.973, 74.323) = 21.518, *p* < 0.0001, partial η^2^ = 0.463) and post-supplementation (F(2.594,64.844) = 11.745, *p* < 0.0001, partial η^2^ = 0.320), with post hoc pairwise comparisons demonstrating that all timepoints between 15 and 90 min differed significantly from the baseline (0 min) timepoint. There was no significant main effect of time or treatment group on glucose total area under the curve (tAUC) (Figure 1B). The mean insulin concentration was not different between supplementation groups (Figure 1C). A main effect of time was noted pre- (F(4.429, 110.734) = 31.75, *p* < 0.0001, partial η^2^ = 0.559) and post- (F(3.553,85.264) = 26.104, *p* < 0.0001, partial η^2^ = 0.521) supplementation with post hoc pairwise comparisons demonstrating that all timepoints between 15 and 120 min differed significantly from the baseline (0 min) timepoint. There was a statistically significant interaction between the intervention and time on insulin tAUC (F(1, 24) = 6.172, *p* < 0.0001, partial η2 = 0.205) with increased insulin tAUC post cherry only (pre: 5872 ± 2656, post: 6601 ± 2564 µU/mL; F(1, 11) = 17.148, *p* = 0.02), but not placebo supplementation (Figure 1D). There was no main effect of condition on insulin tAUC.

**Figure 2:**
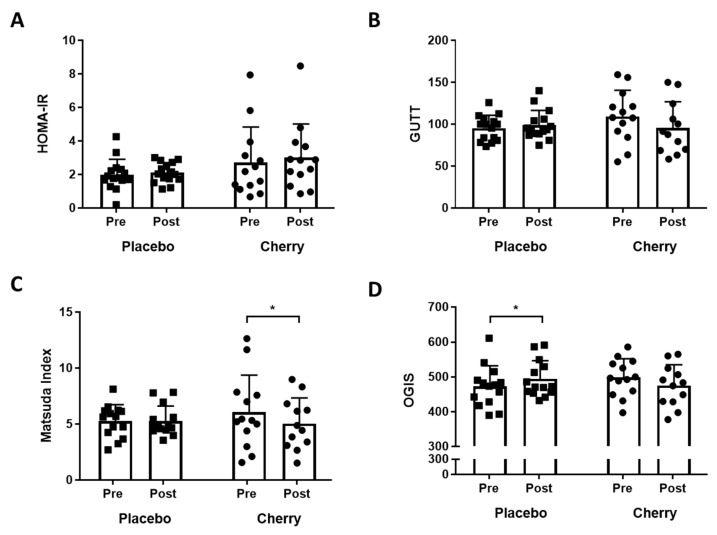
Indices of Glucose Tolerance and Insulin Sensitivity in Placebo and Montmorency Cherry Supplemented Middle-Aged Participants. A total of 28 non-obese middle-aged men and women (40–60 years) consumed a daily dose of Montmorency cherry concentrate (*n* = 13) or an equivalent volume of glucose and energy-matched placebo (*n* = 15) for 4 weeks. An oral glucose tolerance test was conducted pre- and post-supplementation and surrogate measures of insulin sensitivity were calculated. (**A**) HOMA-IR = Homeostatic model assessment insulin resistance; (**B**) the Gutt index of insulin sensitivity; (**C**) Matsuda index; (**D**) OGIS = Oral Glucose Insulin Sensitivity Model. * *p* < 0.05. Missing data points are due to failure of venous access at a necessary timepoint. Black squares = placebo, black circles = cherry.

Surrogate measures of insulin sensitivity are presented in Figure 2. A fasting insulin sensitivity index, HOMA-IR, did not differ with supplementation or time (Figure 2A). For completeness, several dynamic measures of insulin sensitivity are presented. The Gutt index did not differ with supplementation condition or time (Figure 2B). For the Matsuda Index, a significant interaction was found between time and supplementation condition F(1,23) = 5.837, *p* = 0.024, partial η2 = 0.148. Post hoc analysis of simple main effects found that the mean Matsuda index decreased significantly post-MC supplementation (pre 6.1 ± 3.3 vs post 5.0 ± 2.3;F(1,11) = 7.298, *p* = 0.021, partial η2 = 0.399), but not post-supplementation with placebo (pre 5.3 ±1.5 vs post 5.3 ± 1.3; F(1,12) = 0.123, *p* = 0.732, partial η2 = 0.01). For the OGIS, a significant interaction was found between supplementation condition and time F (1, 23) = 7.362, *p* = 0.012, partial η2 = 0.242. Post hoc analyses demonstrated that OGIS increased significantly post-supplementation with placebo (pre 468 ± 60 vs post 497 ± 53 mL/min/m^2^;F(1,12) = 5.565, *p* = 0.036, partial η2 = 0.317) (Figure 2D), but not post-MC supplementation (pre 498 ± 56 vs post 474 ± 60;F(1,11) = 2.483, *p* = 0.143, partial η2 = 0.184).

### 3.2. Metabolic Flexibility

**Figure 3:**
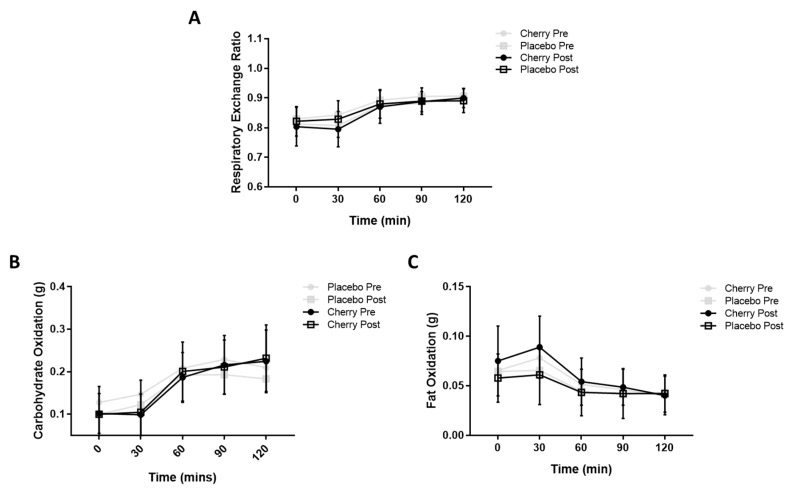
Metabolic Substrate Utilisation During an Oral Glucose Tolerance Test in Placebo and Montmorency Cherry Supplemented Middle-Aged Participants. A total of 28 non-obese middle-aged men and women (40–60 years) consumed a daily dose of Montmorency cherry concentrate (*n* = 13) or an equivalent volume of glucose and energy-matched placebo (*n* = 15). An oral glucose tolerance test was conducted pre- and post-supplementation. Oxygen consumption and carbon dioxide production were measured by pulmonary gas analysis and standard indirect calorimetry equations were applied to calculate respiratory exchange ratio as well as fat and carbohydrate oxidation rates. (**A**) Respiratory exchange ratio; (**B**) Carbohydrate oxidation; (**C**) Fat oxidation.

The RER, carbohydrate oxidation and fat oxidation were quantified throughout the OGTT period. No differences in metabolic flexibility were induced by MC supplementation (Figure 3). The RER did not differ between the placebo and MC conditions either pre- (F(1, 26) = 1.491, *p* = 0.233, partial η2 = 0.054) or post- (F(1, 25) = 0.410, *p* = 0.528, partial η2 = 0.016) supplementation (Figure 3A). MC did not alter carbohydrate or fat oxidation compared to placebo. The main effects of time were noted for the RER, carbohydrate oxidation and fat oxidation, with post hoc analyses revealing that the 30, 60, 90 and 120 min timepoints differed significantly from baseline. Participant energy intake, macronutrient intake and total activity did not differ with time or supplement condition (Supplementary Figure S1). MVPA did not differ over the course of the study, but the placebo supplemented group did significantly less MVPA than their MC supplemented counterparts (F(1, 19) = 8.556, *p* = 0.009) (Supplementary Figure S1).

### 3.3. Plasma Inflammatory Markers

**Figure 4:**
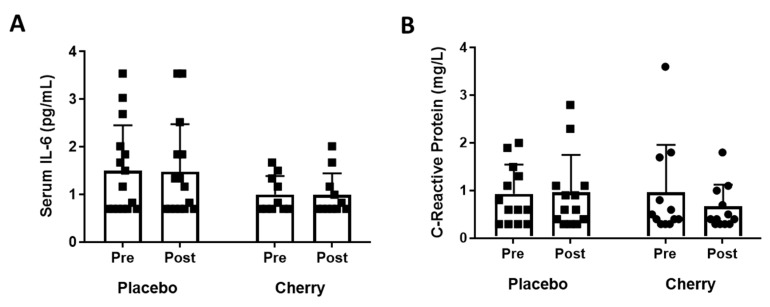
Systemic Inflammatory Burden in Placebo and Montmorency Cherry Supplemented Middle-Aged Participants. A total of 28 non-obese middle-aged men and women (40–60 years) consumed a daily dose of Montmorency cherry concentrate or an equivalent volume of glucose and energy-matched placebo for 4 weeks. IL-6 and CRP were quantified in plasma pre- and post-supplementation. (**A**) Serum IL-6 concentration; (**B**) Serum C-reactive protein concentration. Missing data points are due to values falling >3 SD from the sample mean in participants reporting an acute illness. Black squares = placebo, black circles = cherry.

Serum IL-6 and CRP were measured as markers of systemic inflammatory burden. Where IL-6 absorbance values fell below the sensitivity of the assay, values are reported at the assay sensitivity threshold of 0.7 pg/mL. The main effects of time F(1, 24) = 0.08, *p* = 0.928, partial η2 = 0.000 and supplement F(1, 24) = 2.23, *p* = 0.148, partial η2 = 0.085 were not statistically significant (Figure 4A). Plasma CRP was not altered by supplement condition F(1, 23) = 0.420, *p* = 0.523, partial η2 = 0.018 or by time F(1, 23) = 1.614, *p* = 0.217, partial η2 = 0.066 (Figure 4B).

### 3.4. Gut Microbiome

**Figure 5:**
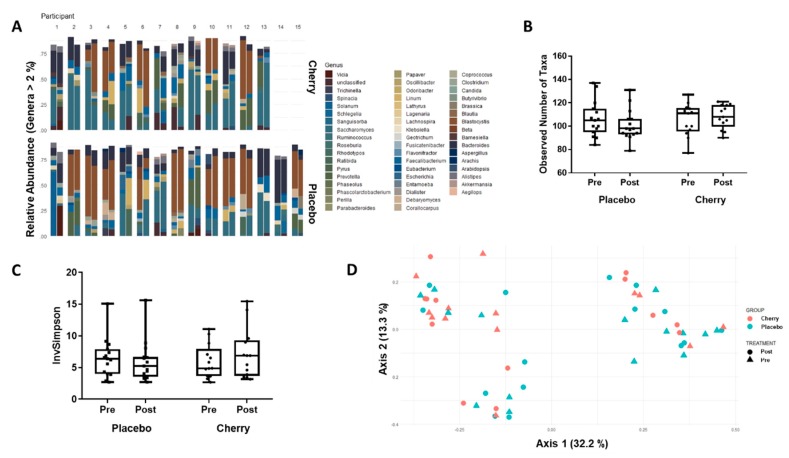
Gut Microbiome in Placebo and Montmorency Cherry Supplemented Middle-Aged Participants. A total of 28 physically inactive and non-obese middle-aged men and women (40–60 years) consumed a daily dose of Montmorency cherry concentrate (*n* = 13) or an equivalent volume of glucose and energy-matched placebo (*n* = 15) for 4 weeks. Faecal samples were collected pre- and post-supplementation, genomic DNA was isolated and the bacterial microbiome was sequenced. (**A**) Relative abundance of genera of abundance >2% of the faecal microbiome. Paired bars represent participant microbiome before (left bar) and after (right bar) the intervention. (**B**) Species richness. (**C**) Species diversity—inverse Simpson Index. (**D**) Principal component analysis.

The dominant gut genera found in participants in this study are described in Figure 5A. Only *Faecalibacterium* represented >2% of the gut microbiome in all participants, with *Bacteroides* present at >2% levels in 26/28 participants. No significant correlation existed between the pre- to post-supplementation change in *Bacteroides* and *Faecalibacterium* abundance and any index of insulin sensitivity (Table 2). The number of observed taxa (species richness) did not differ with time (F(1, 26) = 0.375, p = 0.546, partial η2 = 0.014) or supplementation condition (F(1, 26) = 0.486, *p* = 0.492, partial η2 = 0.018) (Figure 5). Neither did the Simpson Diversity index differ with time or intervention (Figure 5). Species diversity did not differ with time (F(1, 26) = 0.385, *p* = 0.540, partial η2 = 0.015) or supplementation condition (F(1, 26) = 0.80, *p* = 0.779, partial η2 = 0.003) (Figure 5).

PCoA using the Bray–Curtis measure of dissimilarity indicated that the first three axes accounted for 55.5% of the total variation. No significant clustering was observed according to group or treatment. However, the Gap statistic suggested there are at least three clusters (Figure 5). The placebo supplemented group was found to significantly cluster according to habitual physical activity (days per week reporting >30 min activity), but no other outcome measure that we quantified could explain the clustering indicated by the Gap statistic.

## 4. Discussion

Here, we examined the effect of 4 weeks of placebo and MC supplementation on indices of glucose tolerance, insulin sensitivity, metabolic flexibility, systemic inflammation and gut microbiome composition in a population aged 40–60 years. MC supplementation did not alter blood glucose responses to an OGTT compared to placebo. A number of indices of insulin sensitivity were calculated, with some indices indicating no effect of supplementation on fasting (HOMA-IR) or dynamic (Gutt) insulin and glucose responses to supplementation with placebo or MC. However, the Matsuda index and OGIS results are conflicting. The Matsuda index decreased significantly in the MC group post-supplementation, indicating a decrease in insulin sensitivity; this finding reflects the increase in serum insulin tAUC, although notably no corresponding decrease in glucose tAUC was observed. Contrastingly, placebo, but not MC supplementation induced a 6% increase in the OGIS estimate of glucose clearance. For feasibility and cost reasons, we chose an OGTT approach to assessing glucose handling. However, we present several indices of insulin sensitivity in order to present a comprehensive impression of such sensitivity. Meta-analysis of 18 such surrogate measures of insulin sensitivity found that the indices presented here have amongst the strongest pooled correlations to the gold-standard via hyperinsulinaemic euglycaemic clamps (OGIS; r = 0.70 [0.57, 0.80], n = 6), the Matsuda index (r = 0.67 [0.61,0.73], n = 19) and the Gutt index (r = 0.65 [0.60, 0.69], n = 6) [42]. Overall, these results suggest that chronic placebo and MC supplementation with matched supplement carbohydrate content does not alter glycaemic control in this population. Negative correlations have been described between the post-prandial OGTT RER and glucose tAUC as well as the 180-min RER and insulin resistance in older adults with impaired glucose tolerance [45]. Metabolic substrate utilisation did not differ between the placebo and MC conditions at either timepoint; this is perhaps unsurprising, given the lack of a clear difference in glucose handling post-OGTT in our study population. Notably, none of the participants met the diagnostic criteria for impaired glucose tolerance (blood glucose 7.8–11 mmol/L 2 h post a 75 g oral glucose load [46]) at baseline or follow-up. The potential for improvement in glycaemic control might be greater in a population with impaired glucose tolerance or T2D. Indeed, several recent reviews of the evidence for polyphenol augmentation of glucose metabolism have concluded that while many sources of dietary polyphenols show promise for the prevention and management of T2D and glucose intolerance, the diversity in (1) the polyphenolic compounds studied, (2) the doses administered, (3) the duration of such studies and (4) study quality, ensures that no firm conclusions can be drawn [4,47]. Therefore, we consider high-quality studies of the effect of MC and other polyphenol-rich supplements or diets on glycaemic control in such populations to be a research priority. However, restricted intake of refined carbohydrates is recommended in such populations [48]; given 30 mL of MC contains 17.9 g of sugars, such a study may require supplementation with a freeze-dried form of Montmorency cherry supplement. This is particularly pertinent since the small number of human studies that have assessed the effects of polyphenol supplementation on the gut microbiome and found increases in putative anti-inflammatory species (*Lactobacillus* spp [24], *Bacteroides* spp [25] and *Bifidobacterium* spp [24,25,26,27]) have utilised either extracts or powders with low carbohydrate load.

MC supplementation did not alter plasma IL-6 and CRP concentrations compared to placebo. MC-induced reductions in serum IL-6 response to intensive exercise have been described in several studies [14,32,33,49]. However, other studies have shown no effect of cherry supplements on serum markers of inflammation, including IL-6 and CRP [15,50,51]; notably, the exercise protocols in such studies did not induce elevations in any serum inflammatory marker. Pertinently, our study population did not have a substantial degree of baseline systemic inflammation. Despite using a highly sensitive IL-6 assay, 6 placebo-supplemented and 5 MC-supplemented participants fell below the sensitivity threshold of the assay (0.7 pg/mL). This is in broad agreement with published reference values; those aged 19–49 years have a median plasma IL-6 concentration of 1.13 pg/mL, with 25% of this population having a plasma IL-6 concentration of less than 0.76 pg/mL [52]. IL-6 was chosen as previous studies have shown that MC supplementation can reduce serum IL-6 [14,33]. Furthermore, IL-6 is a pleiotropic pro-inflammatory cytokine with complex physiological and pathophysiological roles in ageing [53], insulin resistance [54] and adaptations to exercise [55,56,57]. Similarly, plasma CRP concentrations were low in this study population, with no participant (absent a reported acute illness) recording a value of >4 mg/L. The interquartile range for U.S. adults aged 40–49 years has been calculated as 0.8–4.5 mg/L [58]. It would appear likely that this study population did not have a sufficient systemic inflammatory burden to demonstrate any anti-inflammatory effect of MC. Animal studies of polyphenolic compounds have suggested that they can ameliorate a high systemic inflammatory burden, e.g., grape polyphenols reduced systemic inflammation in high-fat diet-fed mice [59], curcumin reduced hepatic inflammation and macrophage infiltration of white adipose tissue in high-fat diet-induced obese and leptin-deficient ob/ob male mice [60] and polyphenol-rich dendrobium loddigesii extract has been shown to reduce serum IL-6 in db/db Mice. Studies of polyphenol supplementation and systemic inflammation in human clinical populations are limited. A 6-week resveratrol supplementation (500 mg/d) in T2D [61], 3 months of curcuminoids (500 mg/d) plus piperine (5 mg/d) administration in T2D [62] and 4 weeks of cocoa (>400 mg soluble polyphenols) consumption in hypercholesterolaemic adults [63] failed to demonstrate a reduction in systemic inflammation. Participants in our study received a comparatively high bioactive dose (296 mg total anthocyanins and 1040 mg total polyphenols per day), although comparisons between studies using different bioactive substances should be made with caution. There is some precedent for MC reducing inflammation in a clinical population; 6 weeks of tart cherry juice supplementation (>900 mg/d polyphenols) reduced serum CRP in those with Kellgren grade 2–3 osteoarthritis [64]. Inflammation is now thought to be an important aetiological factor in osteoarthritis (OA) [65]. It is clear that further investigation of the anti-inflammatory properties of polyphenol-rich supplements, including MC, is required and that clinical conditions in which inflammation is mechanistically relevant should be targeted to elucidate whether benefit exists.

MC and placebo supplementation did not alter the dominant gut genera for participants. No differences in species richness and diversity were noted and these measures did not display a significant correlation with any index of glycaemic control. Only *Bacteroides* and *Faecalibacterium* abundance was sufficient in a majority of participants to warrant correlation with such indices, with no significant relationships being discovered. Previous human intervention studies suggested that polyphenol consumption increases *Lactobacillus* [24], *Bacteroides* [25] and *Bifidobacterium* [24,25,26]. However, such studies have relied upon methodologies such as bacterial colony culture and morphological identification [26], fluorescence in situ hybridization [24] and RT-qPCR targeting a limited number of bacterial groups [25,27]. Indeed, the abundance of *Lactobacillus* and *Bifidobacterium* in our samples was such that they constituted a small proportion of the gut genera, below the abundance threshold (2%) that is traditionally used for microbiome reporting. Almonacid et al. generated reference ranges for clinically relevant microbial gut genera in 897 adults. After adjustment for the absence of most taxa in a substantial number of individuals is accounted for, the median abundance of *Bifidobacterium* is approximately 0.08%, with 0% (no reads) considered to be the healthy lower limit. For *Lactobacillus*, the median abundance is approximately 0.005% [66]. Indeed, we found that 22/28 participants in this study had no *Lactobacillus* at baseline. Notably, our samples were stored at room temperature for up to 10 months in accordance with the manufacturer’s guidance that samples are stable for up to 2 years in their proprietary nucleic acid preservation solution. However, others have found that rare taxa abundance can be altered by room temperature storage in RNAlater^®^ [67]. It is therefore possible that this altered the abundance of *Bifidobacterium* and *Lactobacillus* in our samples. Our sample size calculations were based on a hypothesised increase in *Bifidobacterium* with MC supplementation. Thus, our study may be underpowered to detect other changes, e.g., microbiome richness and diversity measures. It is also possible that the intervention was not of a sufficient duration to observe a significant change in our outcome measures. The dose of MC chosen was that which has been shown to be effective in enhancing recovery from exercise. However, there remains a dearth of information in the literature regarding the optimal bioactive doses to modify the gut microbiome and glucose handling.

We found that 4 weeks of MC supplementation did not alter glycaemic control or systemic concentrations of IL-6 and CRP in a middle-aged population. We hypothesised, based on previous investigations of other polyphenol-rich supplements that MC may alter the gut microbiome. However, we observed no change in species content, richness or diversity. The middle-aged study population had normal glucose tolerance and no inflammatory burden, and we suggest that future work should focus on clinical populations with inflammatory conditions.

## Figures and Tables

**Table 1:** Participant Baseline Characteristics.

	Placebo	Cherry	*p* Value
Age (yr)	53.2 ± 5.6	48.9 ± 5.4	0.05
Sex	8 M/7 F	8 M/5 F	
Body Mass Index	24.1 ± 2.1	25.8 ± 2.3	0.05
Waist Circumference (cm)	84.6 ± 10.6	86.2 ± 9.4	0.41 ^^^
Habitual Physical Activity (days/week >30 min activity)	3.8 ± 2.0	4.5 ± 1.9	0.38
Flavonoid Intake (mg)	1086 ± 514	1353 ± 535	0.19
Anthocyanin Intake (mg)	26.4 ± 23.2	27.2 ± 16.3	0.59 ^^^
Proanthocyanin Intake (mg)	326 ± 147	310 ± 128	0.77

*p* value represents results of unpaired t test between placebo and cherry conditions, unless denoted by ^^^, where values were non-parametric and a Mann–Whitney U test was used.

**Table 2:** Correlation coefficients for selected genera and indices of insulin sensitivity.

		Insulin tAUC	Glucose tAUC	Matsuda	GUTT	HOMA-IR	OGIS
Bacteroides	r	−0.35	−0.10	0.08	0.07	−0.17	0.03
	95% CI	−0.67 to 0.09	−0.49 to 0.32	−0.33 to 0.47	−0.34 to 0.46	−0.54 to 0.25	0.38 to 0.43
	*p* value	0.10	0.63	0.69	0.73	0.41	0.89
Faecalibacterium	r	−0.05	0.03	0.08	−0.12	0.01	−0.16
	95% CI	−0.44 to 0.37	−0.37 to 0.41	−0.32 to 0.46	−0.48 to 0.29	−0.38 to 0.40	−0.52 to 0.24
	*p* value	0.82	0.89	0.68	0.57	0.97	0.41
Richness	r	−0.03	0.15	−0.16	−0.20	0.06	−0.27
	95% CI	−0.41 to 0.35	−0.25 to 0.50	−0.51 to 0.23	−0.54 to 0.20	−0.33 to 0.44	−0.59 to 0.13
	*p* value	0.87	0.45	0.41	0.31	0.75	0.17
Diversity	r	−0.21	0.04	0.30	0.20	−0.11	0.16
	95% CI	−0.55 to 0.19	−0.35 to 0.42	−0.09 to 0.61	−0.20 to 0.54	−0.47 to 0.28	−0.24 to 0.51
	*p* value	0.29	0.85	0.12	0.31	0.57	0.43

## Supplementary Materials

The following are available online at https://www.mdpi.com/2072-6643/11/5/1063/s1, Figure S1: Energy Intake and Physical Activity in Placebo and Montmorency Cherry Supplemented Middle-Aged Participants.
