# Supplementary material for: Tart Cherry Concentrate Does Not Alter the Gut Microbiome, Glycaemic Control or Systemic Inflammation in a Middle-Aged Population
Source: Nutrients. 2019 May 13;11(5):1063. doi: 10.3390/nu11051063 (PMC6567170; doi:10.3390/nu11051063)
Supplement: Supplementary file 1 [file nutrients-11-01063-s001.pdf]

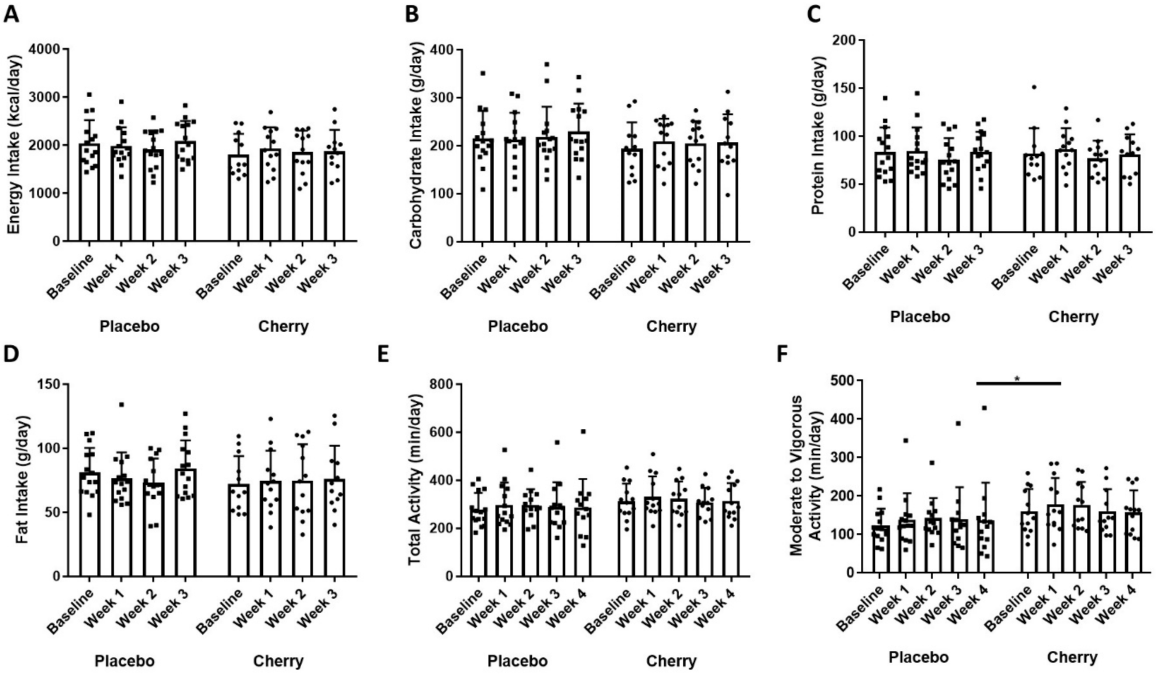

Figure S1. Energy Intake and Physical Activity in Placebo and Montmorency Cherry Supplemented Middle-Aged Participants.
